# Drunkenness

**Published:** 1867-11

**Authors:** 


### HALL'S JOURNAL OF HEALTH.

Vol. XIV.] NOVEMBER, 1867. [No. 11.

**DRUNKENNESS.**—I knew a man whose ships floated on every sea. Every thing he touched turned to gold. Throughout his whole mercantile career he never failed, never suspended, never asked an extension. A large family of children survived him. They were said to be the most beautiful in one of the largest cities of the nation. He himself was the handsomest of men. Commanding in his appearance, courteous in his manner, affectionate in his domestic relations, indulgent to his children, devoted to his wife. During his life he furnished money in the most lavish profusion for family expenditure, never making inquiry as to its disposal. At his death he provided for his wife a munificent income, and left every child rich. In the settlement of his estate, scarcely a dollar was lost. He never was involved in a law-suit, and had but one partner in business, whom he left his sole executor. He had three drawbacks: he was a gourmand; he was never drunk, but was always full of liquor; and habitually made a butt of religion and its ministers. He died before he reached three-score years and ten, of chronic diarrhea, (as most persons do who habitually indulge in highly seasoned food and the finest wines and brandies,) about seven months before the time he had fixed on for retiring from business. The subsequent history of that large family of highly favored children is suggestive. The eldest daughter, of queenly presence and beauty, died an exile from her father's house. Four of her sisters died on the very threshold of beauteous womanhood—one of them in madness; a fifth, by reason of bodily infirmity, is dead to the world; and a son has long been in an asylum, a hopeless idiot Another son survives, a bankrupt, having no business capacity whatever. Another lives, of no promise, and the mother is dead. The terrible lesson here taught is simply this: that the man who makes every day a feast of fat things, and sustains himself by never allowing alcohol to die out of him, except for a few hours in the after-part of the night, must perish prematurely, and can not beget healthy children. Every child this man had was born with a rotten constitution, except the first two, when it may be reasonably supposed he had not completely fallen under the dominion of high-living. It may be well to remark here that four fifths of the idiotic children in a well-conducted asylum for such, were known to be the children of parents one or both of whom indulged in liquor-drinking. I saw a man in a lunatic asylum, an inmate for thirty years, the eldest son of one of the greatest men of our generation, who, up to the time of his marriage, and for a year or two after, indulged freely in whisky-drinking.

A case in point is found in a recent statement of Dr. Hull, superintendent of the Ohio State Asylum:

"A citizen of this State married an intelligent lady, who bore him ten children. After the birth of the first three, the father became intemperate, and during his career as an inebriate, four children were born unto him. He then reformed entirely, and had three others.

"The first three were smart and intelligent, and became useful men and women, and so of the last three. Of the four born to him during his inebriety, two have died in the lunatic asylum, another is there, and the fourth is an idiot! This is not an isolated case. The demonstration is complete and certain, and there is no room left for doubt as to the cause of idiocy and insanity in these cases. Thus an intemperate man or woman transmits a depraved constitution, and an impaired intellect to children, and even to grand-children. The statistics in regard to the idiots of Massachusetts, published a few years since, furnished a volume of proofs to the same general statement. The more this subject is investigated, the more certain it will be shown, that the use of liquors is impairing the health and reason, and shortening the lives, not only of those who drink, but of their descendants."

One of the most eminent politicians of the last thirty years died a sot lately. His son, a clergyman, died soon after, of delirium tremens, and his daughter, the wife of one of the first men of this city, (now dead,) was a slave to the bottle.

Not a dozen years ago a man died who left his daughter a million of money. She shone in foreign courts; but so besotted was she, that her husband was compelled to have an attendant who should never leave her side, for the very instant she was left alone, she would leave the house for the nearest grocery.

An accomplished lady, the wife of one of our leading men, became so addicted to the use of stimulants, that a large part of her time was employed in devising experiments for obtaining any thing that would intoxicate. Finding argument of no avail, the husband, taking advantage of a lucid interval, placed before her the single alternative, either to go to a lunatic asylum, or abandon on the instant and forever all that could intoxicate. She chose the latter, and for the last five years has been an ornament to society. That she will yet die a sot is almost certain.

A drunken man was sent to the penitentiary. His desire for drink was so overwhelming, that, snatching a hatchet, he severed his left wrist, and running to the keeper, called for a bowl of brandy to stanch the blood. Dipping the bleeding stump into the liquid in one instant, he frantically seized the bowl the next, and drank it to the dregs!

One of the most beautiful of women, from Albany, married a New-Yorker. Her wealth, her social position, and naturally fine mind, with genial manners and a large heart, enabled her to command every where, and at once, the respect, the love, and the admiration of all who came in contact with her. She lived but a few blocks from us, and died lately, at twenty-two, of "heart disease," it was said; but the inner circle knew that her love for drink was desperate. Yet so closely was she watched, that the cologne-water of her toilet was the only thing she could have access to, and of that she consumed vast quantities.

It is officially stated, that up to December 31st of the past year, the applications for admission to the Reformatory Inebriate Asylum of the State of New-York, at Binghampton, already approaches very near five thousand names, from all circles of society, and some from foreign lands. Nearly two thousand of these are women from the upper classes; women who, by their position, refinement and culture, reached superior social distinction. And yet all these, by their very application, confess that they find themselves victims to an appetite which they are powerless to resist; confessing that without assistance they feel assured they will sink remedilessly into degradation and a premature grave.

In the light of these facts, the inquiry of a cotemporary becomes most impressive: Could all the daughters of sorrow in our land, whose husbands are habitual inebriates, be gathered together, it would surprise some to discover how many of them were ladies of great delicacy and refinement, brought up amid wealth and luxury, and even amid all the blessings of religion and piety. The common opinion, that drunkenness belongs to the poorer and more degraded of our population, and that it most abounds only in dens and garrets, is, indeed, a very mistaken one. A lady of much intelligence and refinement, reluctantly stated recently, that the wealth of her husband was his bane, as it drew around him a set of fascinating companions, who drew on his purse for suppers and treats, and who would not let him off until his means were exhausted, and he himself was an unconscious and ruined sot. For weeks he would abstain and make great promise of reform, when he would again fall into their hands; and this was repeated until all was lost.

Nor does the habitual use of opium throw a less fatal spell over its helpless, hapless victim. The news comes across the water, that the man whose writings have led captive hundreds of thousands of entranced readers, whose words of burning eloquence have thrilled successive British Parliaments, and whose legal abilities made him Chancellor of the greatest nation on earth—such a man, with such a mind, had not the moral power to break the chains of his enthrallment, and the great novelist, by the use of opium, has fallen into "bodily and mental imbecility," and is forever lost to himself, his country, and the world.

New-Yorkers who were in fashionable society twenty years ago, remember the advent, from a neighboring state, of one of the most accomplished and dazzling beauties of her time. She was followed from one salon to another by crowds of almost crazed admirers, for the brilliancy of her conversation charmed as many as her personal attractions, while her social position was undisputed. She married a person of high position, of great amiability of character, and "the handsomest man in New-York." He soon found that she was addicted to the use of opium—hopelessly so. This so preyed upon his mind, that he committed suicide. Her progress downward became more appalling, and soon the grave claimed her; but not a living soul followed the cartman who conveyed her to her last resting-place.

Within the easy memory of many a New-Yorker, the names of eight clergymen of this city, whose resistless eloquence in the pulpit made them, to many, almost demi-gods, stand to-day before the world as terrible mementoes of the devastative power of the social glass.

The truth is, to be a great orator, a peerless beauty, or the star of the social circle, whether man or woman, is next door to being lost, and the reason of it is patent. They all feel that much is expected of them wherever they go, and a benevolent wish to please, combined with the pride of sustaining a reputation for brilliancy, stimulate them to their highest efforts. But now and then it will happen that the animal spirits are not adequate to the occasion; they feel it, and rather than there should be a failure, strong tea or coffee is resorted to, then the wine, and the brandy, and the pill. As time passes, these become more frequently necessary, and in larger quantities also, until, finally, no effort is made without them. Before they are aware of it, they are utterly unable to overcome the slavish appetite, and are waked up to their misfortune either by dishonor or death.

Within a year, a physician of large practice assured the writer, that although he was not yet forty, he found it absolutely necessary, before he went to see a patient, to bring his mind up to the prescribing point by taking opium.

An eminent lawyer, in our street, not forty-five years old, could not go to his office of a morning, to attend to its ordinary duties, without half a glass of clear brandy. He was considered a brilliant man. None but the few who knew him intimately—not even his wife and children—ever had a suspicion, apparently, that he was a toper; and yet, a year later, he dropped down dead in an out-of-the-way drinking-saloon, "of disease of the heart," the coroner's jury said.

It is thus clear that the professional man, whether physician, lawyer, or clergyman, as well as those who are the charm of society, who are noted and courted for their brilliant powers of conversation, whether belle or beau, are at the very vestibule of ruin when they find themselves taking any stimulant whatever, preparatory to the satisfactory performance of any public, professional, or social duty.

The recently published Custom House tables show that three hundred thousand pounds of opium were imported into this country. Of this amount, reliable data show that only one tenth is used for medicinal purposes. Druggists assure us that the habit of eating opium is rapidly extending among lawyers, physicians, literary men, and ladies, who move in the higher circles of society; and that enormous quantities are used by the manufacturers of patent medicines, and of those poisonous drinks in the saloons, restaurants, coffee-houses, and groggeries, which infest every city and village in the land.

That some means are needed to curtail the use of all that can intoxicate, hear what a late number of the *Irish Quarterly Review* says of the learned and eloquent Dr. Maginn, who might have been prosperous and eminently useful, but whose life was blasted by the wine-cup.

"He now turned for comfort and inspiration to the foul fiend, Brandy, which has been the cause of misery and death to so many men of genius. We regret the errors of Addison and Steele; we sigh at the recollection of poor Moreland, the painter, working at his last picture, with a brush in one hand and a glass of brandy in the other; for he had arrived at that terrible condition, in which reason could only reach him through intoxication; and Maginn, not so fallen as this, sunk deeply. The weary hours of lonely watching brought no resources but that which copious draughts of the liquid could supply.. Health was fading away. The brightest years of life were past forever, and as the dim future lowered, he gazed upon it under the influence of the demon which enthralled the brilliant souls of Addison, of Sheridan, of Charles Lamb, and which sent the once stalwart form of Theodore Hook, a miserable, wretched skeleton, to the grave."

Says a writer in a late number of the *New-York Daily Times:*

"Last Saturday night, in a walk from Nassau street to South Ferry, we had ample food for comment upon the Fourth Commandment. 'Broadway was a perfect hell of drunkenness—a howling, staggering, pandemonium of brutalized men.' The sidewalks were traversed by men in every stage of intoxication, reeling to and fro like ships in a storm. The air was ladened with snatches of drunken songs, fragments of filthy language, or incoherent shouts from those who were too drunk to articulate. Drunkenness in every dark lane and alley, only discovered by its disgusting ravings. Drunkenness in the wide lamp-lit streets, staggering along with swimming heads, paralyzed limbs, and countenance of imbecile sensuality. Drunkenness in the kennel, stentoriously respiring its fetid breath. Drunkenness clinging to the lamp-posts. Drunkenness coiled upon the door-steps, waiting to be robbed or murdered. Drunkenness screaming on the tops of solitary omnibuses, or hanging half out of the windows of belated hackney-cabs, and disturbing the night with incoherent melodies. Drunkenness walking apparently steady along, idiotically to itself, and thickly rehearsing the drunken jokes and drunken songs, the indecencies that adorn the convivial meeting it has just left. Drunkenness waiting at the ferries, snoring on benches, quarreling with its drunken company, or falling off the edge of the pier into the water, and being fished out half sober."

With such facts before us, let every good citizen, let every man, woman, and child in the nation, feel that there is no certain escape from the remorseless despotism of drunkenness, except in the practice of total abstinence from every thing which can intoxicate, whether it be a liquid, a solid, or a gas; for the fumes of chloroform are becoming the resort of the nervous, the dyspeptic, and the hapless victim of *ennui* and idleness. As to the use of wines, beers, brandies, cider, opium, and tobacco, the only infallible guarantee from a wasted life, an early death, the gutter, or the madhouse, is in obeying the counsel of the inspired volume: "TOUCH NOT, TASTE NOT."

